# Supplementary material for: Baobab-Fruit Shell and Fibrous Filaments Are Sources of Antioxidant Dietary Fibers
Source: Molecules. 2022 Aug 29;27(17):5563. doi: 10.3390/molecules27175563 (PMC9457770; doi:10.3390/molecules27175563)
Supplement: Supplementary file 1 [file molecules-27-05563-s001.zip › molecules-1853273-supplementary.pdf]

SUPPLEMENTARY MATERIALS

Baobab-Fruit Shell and Fibrous Filaments Are Sources of Antioxidant Dietary Fibers

Manuela Flavia Chiacchio [1], Silvia Tagliamonte[1], Attilio Visconti[1], Rosalia Ferracane[1], Arwa Mustafa [2] and Paola Vitaglione [1,*]

[1] Department of Agricultural Sciences, University of Naples "Federico II", 80055 Portici, Italy
[2] ARWA Foodtech AB, 22363 Lund, Sweden
* Correspondence: paola.vitaglione@unina.it

Figure S1: Extracted Ion Chromatograms (XIC) of baobab pulp (A), shell (B), fibrous filaments (C) and seeds (D) confirmed by HPLC-MS/MS

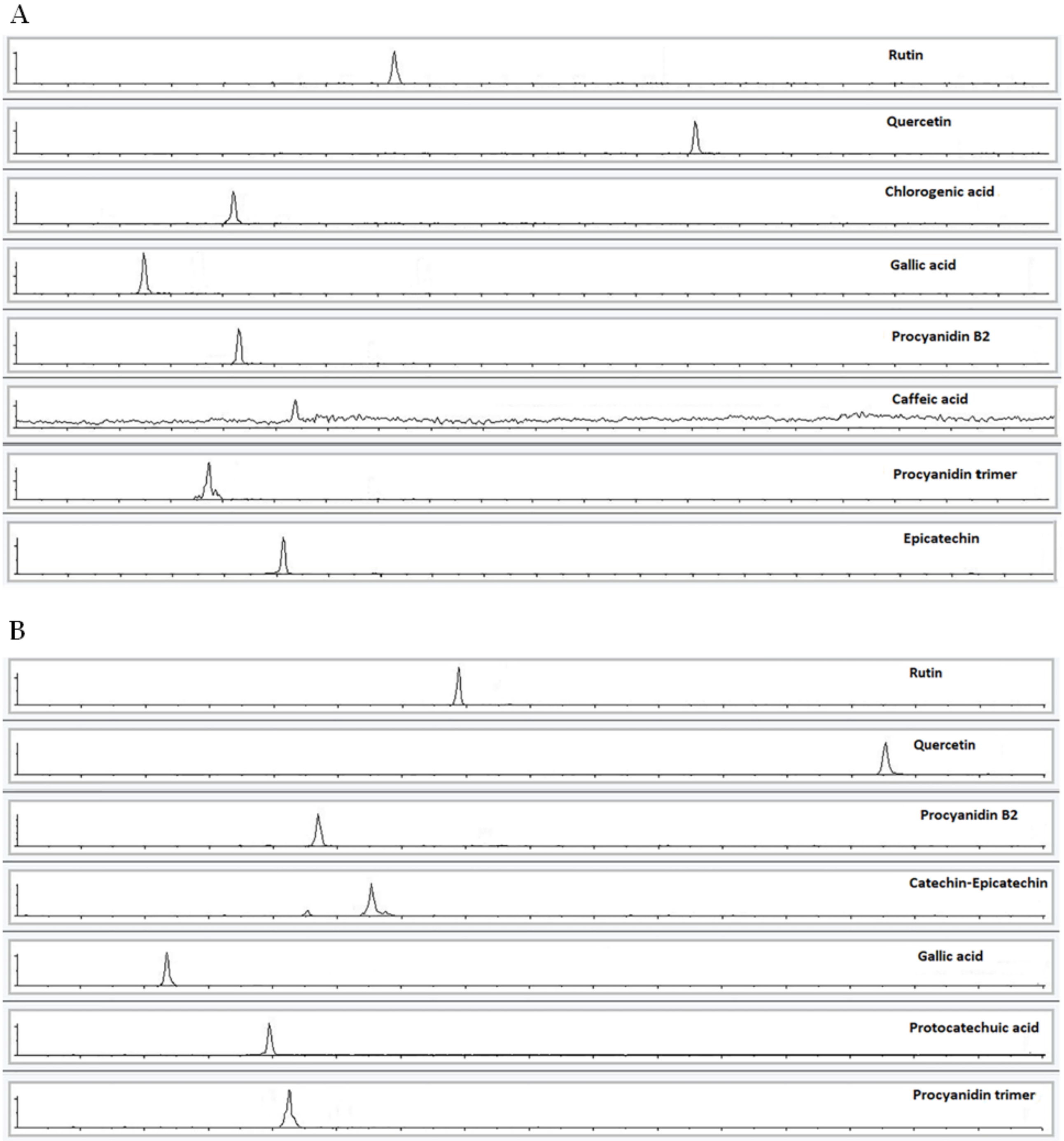

C

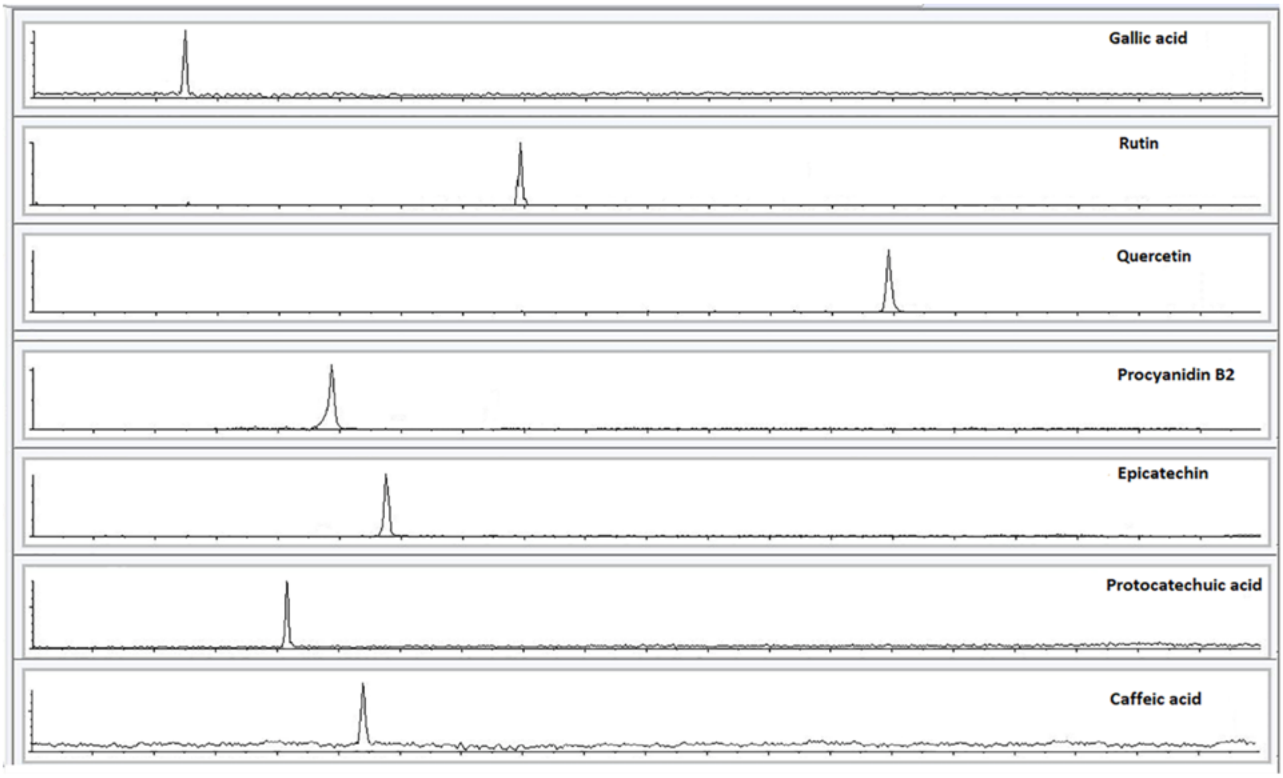
Gallic acid
Rutin
Quercetin
Procyanidin B2
Epicatechin
Protocatechuic acid
Caffeic acid

D

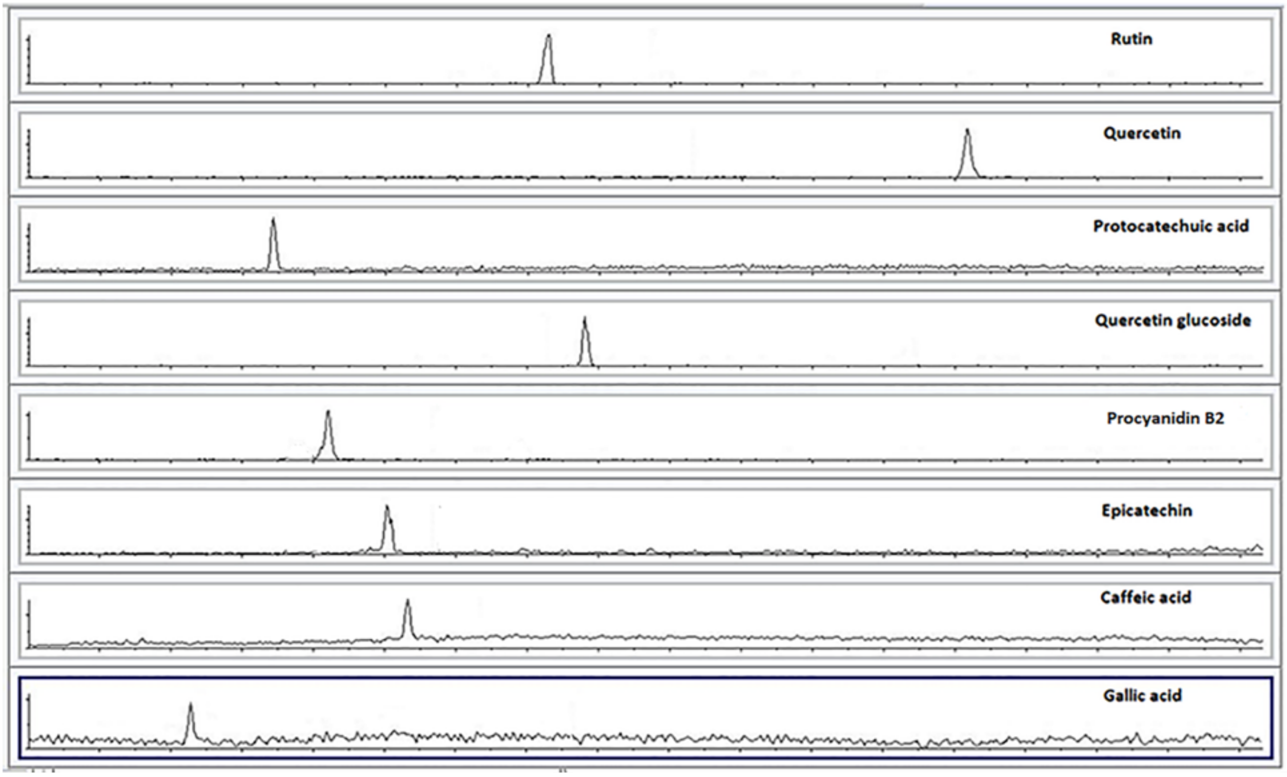
Rutin
Quercetin
Protocatechuic acid
Quercetin glucoside
Procyanidin B2
Epicatechin
Caffeic acid
Gallic acid
